# Transcatheter Aortic Valve Replacement (TAVR)-Induced Hemodynamically Unstable Supraventricular Tachycardia in an Elderly Patient With a Complex Cardiovascular History

**DOI:** 10.7759/cureus.83846

**Published:** 2025-05-10

**Authors:** Joud Fahed, Abdul Mohammad, Rehman Afraz, Danhue Moodie, Claudia Georges

**Affiliations:** 1 Internal Medicine, Ascension St. Agnes Hospital, Baltimore, USA; 2 Internal Medicine, Ross University School of Medicine, Miramar, USA; 3 Internal Medicine, MedStar Franklin Square Medical Center, Baltimore, USA

**Keywords:** atrioventricular nodal reentry tachycardia, electrophysiologic study, radiofrequency ablation, supraventricular tachycardia with aberrancy, trans aortic valve replacement

## Abstract

Transcatheter aortic valve replacement (TAVR) is an important alternative to surgical aortic valve replacement (SAVR) as it reduces stay length and complication rates. Despite this apparent advantage, clinicians must still be aware of these complications. In this paper, we present a case of an elderly female who had an ICD placed years before presentation, presenting with wide complex tachycardia weeks after the TAVR procedure. In this case, the differentiation between ventricular tachycardia and supraventricular tachycardia with aberrancy proved to be a diagnostic challenge. This case highlights an arrhythmia-related complication stemming from TAVR and the utility of the Implantable cardioverter defibrillator (ICD) as an important diagnostic tool in differentiating these rhythms and confirming the temporal relationship between this arrhythmia and the valve replacement procedure.

## Introduction

Transcatheter aortic valve replacement (TAVR) is a revolutionary intervention that allows for the minimally invasive replacement of pathological aortic valves, thereby reducing debilitating symptoms such as dyspnea, chest pain, and fatigue. The primary advantage is reduced risk of complications and length of hospital stay compared to the open surgical approach to aortic valve replacement. Although the benefits gained from this procedure are immense, there are common side effects that should be noted. These complications include arrhythmia such as high-degree AV block and new-onset atrial fibrillation (AF), as well as vascular complications related to transarterial implantation [1,2]. In a meta-analysis of 65 studies encompassing 43,506 patients, supraventricular arrhythmias were reported in only 29 patients compared to 182 cases of bradyarrhythmias, highlighting the rarity of supraventricular tachycardias (SVTs) following TAVR [2]. In this case, we highlight a new-onset wide complex tachycardia, which was later identified to be an SVT with aberrancy. This case highlights two key points: first, the occurrence of a rare post-procedural arrhythmia following TAVR; and second, the diagnostic challenge of accurately identifying the arrhythmia despite the application of established clinical algorithms such as the Brugada and Vereckei criteria.

## Case presentation

This case involves an 84-year-old female with a complex cardiac history, including coronary artery disease status post coronary artery bypass grafting (CABG) and cardiac stent placement, ischemic cardiomyopathy with a reduced ejection fraction of 25%, and a dual-chamber automatic implantable cardioverter-defibrillator (ICD) placed for primary prevention. Before her presentation at our hospital, the patient was admitted for acute decompensated heart failure. During that admission, an echocardiogram revealed severe aortic stenosis. Following appropriate evaluation, she was deemed a suitable candidate for transcatheter aortic valve replacement (TAVR), which was subsequently performed. The procedure was uneventful, and she was discharged with a scheduled outpatient follow-up.

**Figure 1:**
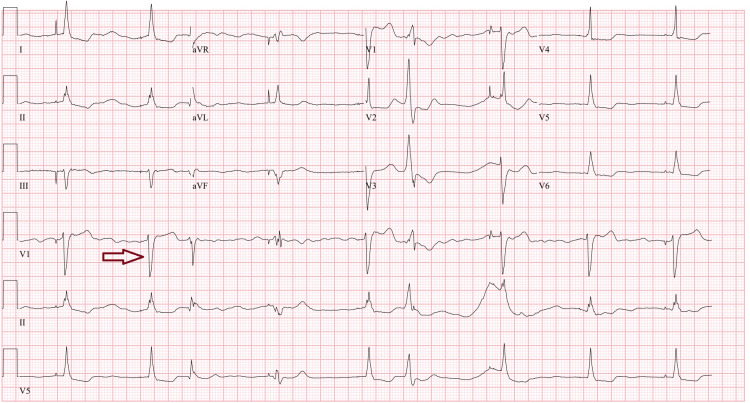
ECG showing junctional rhythm upon arrival to the emergency department after receiving amiodarone in the field. An example of a junctional beat is identified by the red arrow.

Approximately two weeks after the procedure, the patient developed progressive dyspnea, orthopnea, and lower extremity edema, now accompanied by recurrent syncopal episodes. She reported no ICD shocks during this period. Emergency medical services were contacted, and the patient was found to be hypotensive, with a blood pressure as low as 64/40 mmHg, and hypoxic, with an oxygen saturation of 84% on room air. A cardiac monitor was attached, and a wide-complex tachycardia (WCT) with a heart rate around 160 bpm was noted. Synchronized cardioversion was attempted three times, with escalating doses starting at 100 joules, then 200 joules, and finally 300 joules. The patient also received a 150 mg bolus of amiodarone in the field. Following these interventions, there was some slowing of the ventricular rate. Upon arrival at the hospital, her systolic blood pressure was 71/54 mmHg. An ECG in the ED revealed a junctional rhythm with a heart rate of 55 bpm (Figure 1). Due to her hypotension, she was admitted to the ICU. Norepinephrine and an amiodarone drip were subsequently initiated.

The following day, norepinephrine and amiodarone were successfully weaned and discontinued, and she was downgraded to the telemetry unit. However, on the unit, a rapid response was called due to persistent hypotension and tachycardia. Upon review of the telemetry strip, wide complex tachycardia was noted. The patient was initially given 5 mg IV metoprolol, with no improvement. She then received an amiodarone bolus and 100 mg of lidocaine, after which her heart rate dropped to the 40s, and a change in mental status was observed. Post-intervention, the patient appeared more confused and slightly lethargic. In response, she was administered 1 mg of atropine and a small bolus of epinephrine, which resulted in an increase in heart rate and a return to baseline mental status. She was readmitted to the ICU for continued care.

**Figure 2:**
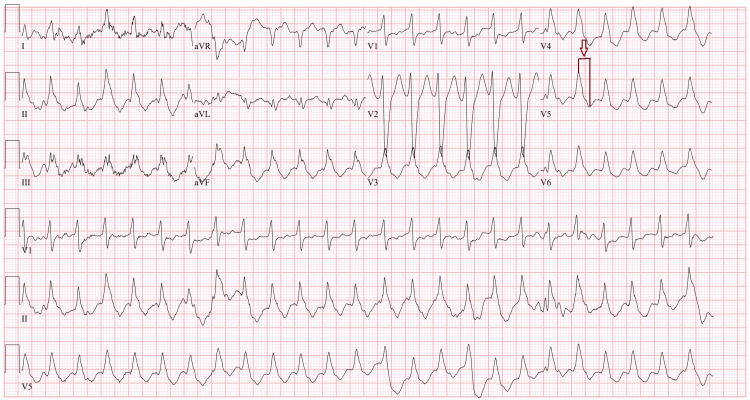
ECG showing wide complex tachycardia with R-S interval > 100 ms (red arrow).

An ECG was performed during the episode of wide complex tachycardia, which showed a wide QRS complex of 166 ms, a heart rate of 163 bpm, a change in axis in lead V2, and a dominant R wave in AVR. These findings, along with the prolonged QRS duration greater than 140 ms, suggested ventricular tachycardia (VT) based on the Brugada and Vereckei criteria (Figure 2).

**Figure 3:**
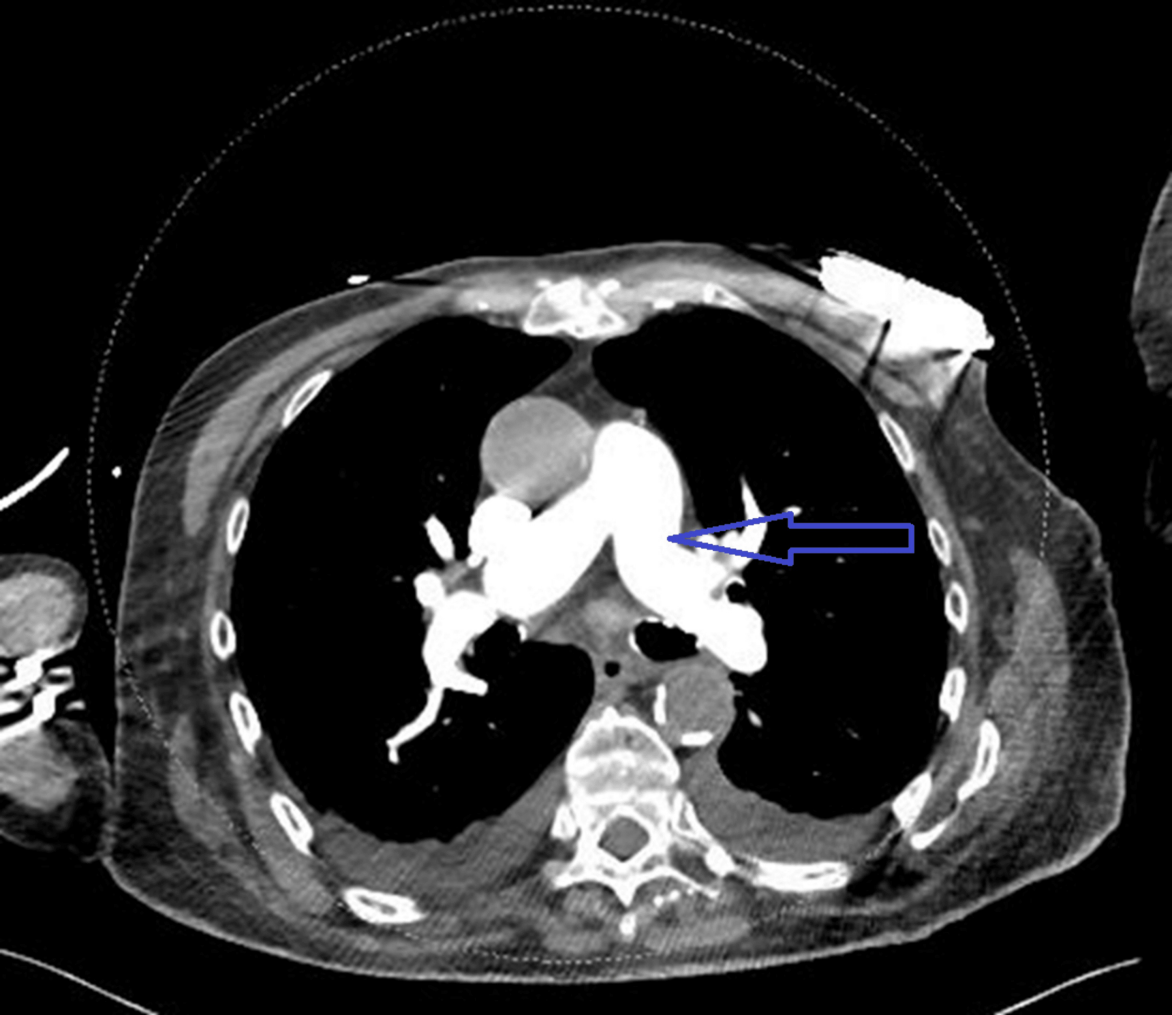
Computed tomography angiography of the chest showing absence of pulmonary embolism.

In addition to the ECG findings, the patient's lab results showed an elevated Troponin I of 1,108 ng/L (at peak), suggestive of myocardial injury, which we suspected to be a type 2 non-ST-segment elevation myocardial infarction (NSTEMI). Her potassium was 3.0 mEq/L, creatinine was elevated at 1.9 mg/dL, magnesium was normal at 2.1 mg/dL, and calcium was mildly low at 8.0 mg/dL, all of which may have been a nidus for arrhythmic activity (Table 1). A subsequent echocardiogram revealed a significantly reduced ejection fraction of 25%-30%, severe diffuse hypokinesis, and moderate mitral regurgitation, consistent with prior history of advanced ischemic cardiomyopathy (Video 1). A CT angiogram of the chest showed no evidence of pulmonary embolism, ruling out this potential cause for the patient’s hemodynamic instability (Figure 3).

**Table 1:** Pertinent blood test results and laboratory reference ranges.

Test	Result	Reference range
Troponin	1,108 ng/L	0-14 mg/dL
Potassium	3.0 mEq/L	3.5- 5 mEq/dL
Magnesium	2.1 mg/dL	1.6-2.6 mg/dL
Calcium	8.0 mg/dL	8.4-10.2 mg/dL

**Video 1:** Echocardiogram performed during admission showing a reduced ejection fraction of 25% to 30%.

**Figure 4:**
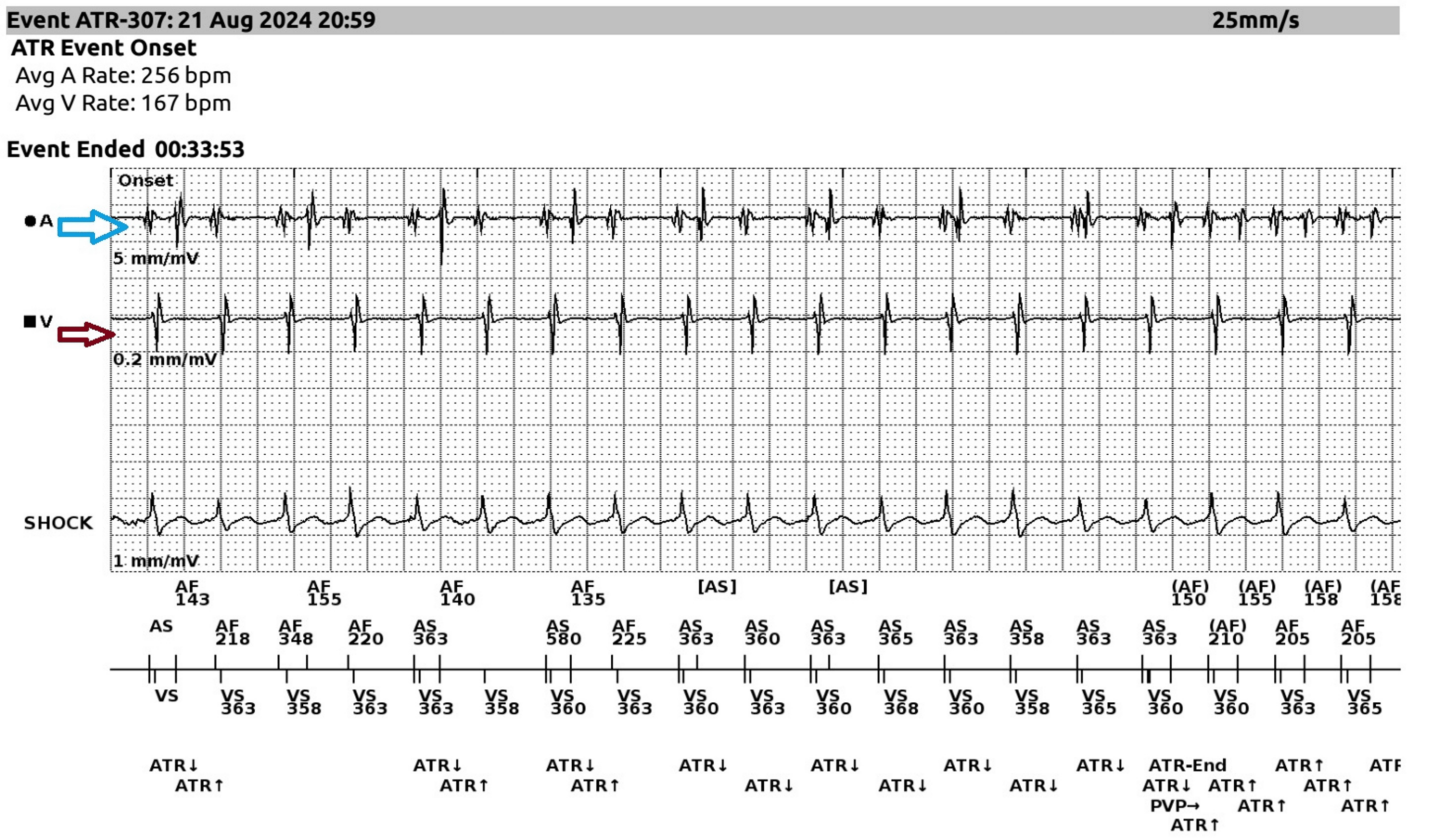
ICD interrogation revealing atrial sense on the atrial EGM (blue arrow) preceding ventricular sense on the ventricular EGM (red arrow), indicating an atrial source of the arrhythmia. EGM, electrogram; ICD, implantable cardioverter defibrillator; ATR, atrioventricular reentrant tachycardia

Following live ICD interrogation (Figure 4), the arrhythmia, initially presumed to be VT, was found to be atrioventricular nodal reentrant tachycardia (AVNRT) with aberrancy. The ICD, programmed in dual-chamber mode (DDD) with sensing and pacing capabilities, recorded atrial sense (A-sense) followed by ventricular sense (V-sense), indicating an atrial origin. This finding prompted further electrophysiological (EP) study evaluation. After the ICD interrogation, amiodarone was discontinued, and the patient was transferred to another hospital for further EP study.

During the electrophysiology evaluation, catheters were advanced to the His region, right ventricular apex, and coronary sinus. The baseline rhythm was recorded as sinus with a cycle time of 1250 ms. Key intervals were noted: the AH interval was 114 ms, the HV interval was 73 ms, the QRS interval was 146 ms, and the QT interval was 463 ms. Ventricular incremental pacing did not show any retrograde conduction to the atrium, ruling out VT. The absence of retrograde conduction and a dominant ventricular pacemaker further classified the arrhythmia as atypical AVNRT with aberrancy, as suspected based on the ICD interrogation. The EP study also identified a slow pathway, which was subsequently targeted for empiric ablation. Atrial extrastimulus testing was performed but was unsuccessful in inducing any atrial arrhythmias. During the ablation procedure, a minimal number of junctional beats were induced despite extensive ablation. Atrial fibrillation was also noted on interrogation during the EP study, so she was started on metoprolol succinate for rate control and amiodarone 200 mg p.o. (two tabs twice daily) for 10 days, followed by one tab daily.

## Discussion

TAVR is a minimally invasive procedure commonly used to replace pathologic aortic valves, particularly in elderly patients with severe aortic stenosis who are not candidates for open heart surgery. The procedure involves inserting a balloon catheter through an artery, usually via the femoral artery, and guiding it to the problematic aortic valve. Once in position, the catheter deploys a new valve to replace the damaged valve, with echocardiography guidance to monitor the valve's proper placement and function. After the procedure, patients are closely monitored for complications such as bleeding, valve patency, and the onset of new arrhythmias or other related issues [1].

In addition to mechanical valve issues, end-organ damage, and bleeding complications, the development of new arrhythmias is a noteworthy concern following TAVR. These arrhythmias are often the result of mechanical damage to the heart’s conduction system. One of the most commonly observed complications is new-onset AF, which has been well documented in the literature. A meta-analysis of 65 studies (43,506 patients) found that approximately 25% of patients developed AF within two years after undergoing TAVR [2]. Moreover, it is estimated that 10%-18% of patients who undergo TAVR may ultimately require a new automatic ICD due to the emergence of arrhythmias following the procedure [3].

SVT is a relatively uncommon complication following TAVR, with only 29 cases documented in a meta-analysis of 43,506 patients, highlighting its low incidence. Among these cases, the incidence of AVNRT with aberrancy in the context of TAVR is even less well-defined in the literature [2].

SVT refers to arrhythmias originating above the bifurcation of the bundle of His, typically from atrial or AV nodal tissue. AVNRT, a common form of SVT, is characterized by reentry within the AV node. When AVNRT occurs with aberrancy, conduction through the His-Purkinje system is delayed or premature, leading to a widened QRS complex [4].

Differentiating AVNRT with aberrancy from VT is crucial, as both can present with wide QRS complexes. The Brugada and Vereckei criteria help distinguish the two by evaluating QRS morphology, AV dissociation, response to vagal maneuvers/adenosine, R-wave peak time in Lead II, and the clinical context [5]. These criteria will be discussed further in the context of our case.

AVNRT results from dyssynchrony between the slow and fast pathways near the AV node. The slow pathway arises posteroinferiorly to the compact AV node, along the tricuspid annulus, while the fast pathway is anterosuperior near Koch's triangle [5]. AVNRT is classified into typical and atypical forms. In typical AVNRT, the slow pathway conducts anterogradely, and the fast pathway retrogradely, producing a short RP interval on the EKG. In atypical AVNRT, the pathways are reversed, with the fast pathway conducting anterogradely and the slow pathway retrogradely, resulting in a long RP interval [6].

Nawata et al. showed that in fast-slow AVNRT, the retrograde slow pathway is in the posterior septum, while the anterograde fast pathway is in the anterior septum [7]. Identifying the form of AVNRT is essential for guiding treatment.

To understand conduction disturbances after TAVR, it's crucial to recognize the location of the conduction system relative to the aortic valve. The AV bundle is located at the apex of Koch's triangle, formed by the coronary sinus orifice, the tendon of Todaro, and the septal leaflet of the tricuspid valve. As the AV bundle enters the membranous septum, it becomes the bundle of His, with the left bundle branch branching off inferiorly and passing through the right coronary and non-coronary leaflets. The triangle of Koch is critical in TAVR procedures, as damage to the AV bundle here is a proposed mechanism for new-onset arrhythmias after TAVR. Anatomical variations, such as a right-sided AV bundle in 50% of individuals and a left-sided bundle in 30%, can increase the risk of conduction disturbances, particularly in patients with a short membranous septum [8].

Two mechanisms are proposed for TAVR-induced AVNRT: mechanical compression and inflammation. Elevated pressure from the TAVR stent can compress conduction fibers between the aortic annulus and the AV node, disrupting conduction and potentially generating reentrant currents in Koch's triangle. This compression, possibly exacerbated by pre-dilation and catheter insertion, can also be worsened by inflammation and edema during the implantation process [9,10]. The resultant anatomical changes and localized inflammation can alter conduction pathways, fostering SVT or AVNRT.

Detection and classification of AVNRT with aberrancy versus VT can be challenging due to overlapping EKG findings. When our patient presented, the arrhythmia was initially classified as VT based on the Brugada and Vereckei criteria. The EKG showed a wide QRS complex (166 ms), a heart rate of 163 bpm, and an axis change in V2. The RBBB pattern in leads V1-V3 and QRS duration >160 ms further supported a VT diagnosis. Elevated Troponin I and the patient's ischemic cardiomyopathy history raised concern for a ventricular origin.

In line with the Brugada and Vereckei criteria, the RBBB morphology in leads V1-V3 and the wide QRS duration (>160 ms) were suggestive of VT, with the morphology criteria showing a sensitivity of 99% and specificity of 97%. Additionally, there was AV dissociation and the R-to-S interval in lead V1 was >100 ms, which, according to the criteria, is indicative of VT with a sensitivity of 66% and specificity of 98% (Figure 2).

However, the final diagnosis was clarified through ICD interrogation and an electrophysiological (EP) study. During the EP study, ventricular stimulation revealed no retrograde conduction to the atrium, ruling out VT, as there was no retrograde transmission and no dominant ventricular pacemaker. This led to the conclusion that the arrhythmia was atypical AVNRT with aberrancy. The EP study also identified a slow pathway, which was subsequently targeted for empiric ablation. The combination of ICD interrogation and EP study findings allowed for a confident reclassification of the arrhythmia as atypical AVNRT with aberrancy, emphasizing the importance of these diagnostic tools in managing complex arrhythmias in the context of TAVR.

One of the most effective treatments for AVNRT with aberrancy is electrophysiology catheter ablation, where cardiac mapping and radiofrequency ablation are used to modify or eliminate the conduction of the slow pathway at the posterior septum near the coronary sinus ostium. Unfortunately, in our patient, the arrhythmia could not be reproduced during the electrophysiology study due to difficulty identifying any junctional beats, even after extensive ablation near this area. As a result, pharmacologic management was pursued, with amiodarone for rate and rhythm control and apixaban for thrombosis prevention. Despite these measures, the arrhythmia recurred, leading to the patient’s second hospitalization. After adjusting the ICD parameters, the patient's AVNRT with aberrancy was expected to be autonomously corrected, reducing the need for further hospitalizations.

Given the non-reproducible arrhythmia in the electrophysiology study and the initial misdiagnosis of VT, it was the dual-chamber ICD that ultimately allowed us to accurately diagnose AVNRT with aberrancy, specifically the atypical form. The ICD interrogation not only clarified the diagnosis but also exemplified the best-case scenario for using these devices in uncovering complex arrhythmias. The detailed data provided by the ICD, in combination with the findings from the electrophysiology study, were crucial in distinguishing AVNRT with aberrancy from other potential diagnoses, including VT. The ICD's interrogation data confirmed that no arrhythmia had been present before the TAVR procedure, which helped rule out any underlying arrhythmia. Although the patient already had an ICD due to prior ischemic cardiomyopathy, this device proved invaluable in diagnosing the new-onset arrhythmia post-TAVR. The evolution of ICD technology is making these devices increasingly essential for managing such cases and enhancing our ability to treat a wide range of arrhythmias, offering better diagnostic precision and tailored interventions.

## Conclusions

This case highlights a rare but potentially fatal complication following TAVR due to the risk of hemodynamic instability. Often misdiagnosed as ventricular tachycardia, the complexity of identifying AVNRT with aberrancy, coupled with its infrequent occurrence post-TAVR, underscores the need for more accurate diagnostic algorithms. The use of ICDs is also important in differentiating AVNRT from VT, offering a valuable tool for accurate diagnosis and appropriate management, especially in complex cases like this. Future research should focus on refining TAVR techniques to avoid mechanical or ischemic damage to the anatomy near Koch’s triangle, a critical area involved in the conduction system. Additionally, developing predictive models to identify patients at higher risk for post-TAVR arrhythmic complications would be valuable. Factors such as patient's age, type of cardiomyopathy, and prior conduction disease would be important factors that could be included in this model.
